# Structural insights into the assembly of the 30S ribosomal subunit *in vivo*: functional role of S5 and location of the 17S rRNA precursor sequence

**DOI:** 10.1007/s13238-014-0044-1

**Published:** 2014-03-28

**Authors:** Zhixiu Yang, Qiang Guo, Simon Goto, Yuling Chen, Ningning Li, Kaige Yan, Yixiao Zhang, Akira Muto, Haiteng Deng, Hyouta Himeno, Jianlin Lei, Ning Gao

**Affiliations:** 1 Ministry of Education Key Laboratory of Protein Sciences, Center for Structural Biology, School of Life Sciences, Tsinghua University, Beijing, 100084 China; 2 Department of Biochemistry and Molecular Biology, Faculty of Agriculture and Life Science, Hirosaki University, Hirosaki, 036-8561 Japan

**Keywords:** RsgA, RbfA, ribosome assembly, cryo-EM, quantitative mass spectrometry

## Abstract

The *in vivo* assembly of ribosomal subunits is a highly complex process, with a tight coordination between protein assembly and rRNA maturation events, such as folding and processing of rRNA precursors, as well as modifications of selected bases. In the cell, a large number of factors are required to ensure the efficiency and fidelity of subunit production. Here we characterize the immature 30S subunits accumulated in a factor-null *Escherichia coli* strain (*∆rsgA∆rbfA*). The immature 30S subunits isolated with varying salt concentrations in the buffer system show interesting differences on both protein composition and structure. Specifically, intermediates derived under the two contrasting salt conditions (high and low) likely reflect two distinctive assembly stages, the relatively early and late stages of the 3′ domain assembly, respectively. Detailed structural analysis demonstrates a mechanistic coupling between the maturation of the 5′ end of the 17S rRNA and the assembly of the 30S head domain, and attributes a unique role of S5 in coordinating these two events. Furthermore, our structural results likely reveal the location of the unprocessed terminal sequences of the 17S rRNA, and suggest that the maturation events of the 17S rRNA could be employed as quality control mechanisms on subunit production and protein translation.

**Electronic supplementary material:**

The online version of this article (doi:10.1007/s13238-014-0044-1) contains supplementary material, which is available to authorized users.

## Introduction

Fast growing bacteria have a tremendous demand for the ribosome so as to produce proteins required for various cellular activities in a timely manner. In *Escherichia coli*, there are around 30 known assembly factors *in vivo*, and some of them possess well-defined enzymatic functions, such as rRNA modification and processing. Genetic perturbation of different assembly factors confers similar phenotypes in general, often characterized by accumulation of rRNA precursors and immature assembly intermediates [reviewed in (Shajani et al., 2011)]. However, further functional elucidation of assembly factors in molecular detail has been staggered, mainly due to three reasons. The first is that translation is such a fundamental process that the interference of ribosome assembly by genetic manipulations of assembly genes often causes disparate secondary cellular disorders. The second is that the assembly process is highly efficient *in vivo*, and most factors play important, but non-essential roles, which makes it impossible to isolate factor-specific intermediates for functional assays. As a result, assembly intermediates from factor-null strains are in principle a heterogeneous set of presumably related intermediates. The last is that the assembly of ribosomal proteins is highly coupled with rRNA maturation, such as rRNA folding, modification and processing (Shajani et al., 2011), and disruption of a single assembly event might elicit a cascade of assembly defects.

Nevertheless, the *in vitro* reconstitution of ribosomal subunit free of assembly factors has remained to be a classical biochemical system for decades, with a focus on studying the binding interdependence among ribosomal proteins [for examples, see (Grondek and Culver, 2004; Mizushima and Nomura, 1970; Rohl and Nierhaus, 1982)] and metastable rRNA conformational transitions [for examples, see (Calidas and Culver, 2011; Holmes and Culver, 2004, 2005; Powers et al., 1993; Ramaswamy and Woodson, 2009; Stern et al., 1989)] during the assembly process. More recently, time-resolved techniques from pulse-labeling based quantitative mass-spectrometry (QMS) (Mulder et al., 2010; Talkington et al., 2005) and synchrotron X-ray footprinting (Adilakshmi et al., 2008) have further generated a large amount of real-time data for various protein binding and rRNA folding events in the process of the *in vitro* 30S subunit assembly. These kinetic data, together with previous knowledge, have stitched a general picture that the assembly of the 30S subunit *in vitro* is a highly branched process, featured with interconnected parallel assembly pathways (Mulder et al., 2010), and the rRNA folding is tightly coupled with protein binding, displaying an apparent “induced fit” strategy (Adilakshmi et al., 2008).

With a wealth of *in vitro* information at hand, in recent years two complimentary approaches were taken to tackle the *in vivo* 30S assembly process. One is to quantify ribosomal protein levels in presumably different assembly intermediates accumulated *in vivo* (from genetically or chemically perturbed strains) through QMS (Clatterbuck Soper et al., 2013; Guo et al., 2013; Jomaa et al., 2011; Leong et al., 2013; Sykes et al., 2010). The other is to use structure-probing methods to investigate rRNA conformations of these *in vivo* intermediates (Clatterbuck Soper et al., 2013; Guo et al., 2013; Jomaa et al., 2011; Leong et al., 2013; Roy-Chaudhuri et al., 2010). Through the integration of the compositional and structural data with the known protein binding interdependences described in the Nomura map (Mizushima and Nomura, 1970), as well as with the roughly determined binding orders of ribosomal proteins (Chen and Williamson, 2013), it starts to become practical to dissect these *in vivo* data to identify rate-limiting steps of the 30S assembly and to uncover molecular roles of assembly factors and ribosomal proteins.

Previously, we have constructed an *E. coli* mutant strain (Guo et al., 2013), in which genes of two assembly factors, RbfA and RsgA, are deleted. RbfA is a cold-shock protein (Jones and Inouye, 1996) involved in the maturation of the 30S subunit (Bylund et al., 1998; Dammel and Noller, 1995; Goto et al., 2011; Xia et al., 2003). RbfA binds to the neck of the 30S subunit (Datta et al., 2007) and its function was genetically linked to the maturation of the 5′ end of the 16S rRNA (Dammel and Noller, 1995). RsgA is a 30S subunit-dependent GTPase also involved in the maturation of the 30S subunit (Daigle and Brown, 2004; Himeno et al., 2004). Importantly, RsgA is one of the few examples among all assembly factors whose molecular role has been biochemically established, which is to promote the timely release of RbfA from the mature or nearly mature 30S subunit in a GTP-dependent manner (Goto et al., 2011; Guo et al., 2011). Disruption of these two genes simultaneously confers phenotypes similar to those of single gene disruptants (Bylund et al., 1998; Dammel and Noller, 1995; Himeno et al., 2004; Jomaa et al., 2011; Xia et al., 2003), such as accumulation of immature 30S subunits and the 17S rRNA precursor in the cell (Goto et al., 2011; Guo et al., 2013).

In the present work, we characterize the immature 30S subunits isolated from this *∆rsgA∆rbfA* strain. The immature intermediates were prepared using two different buffers varying in salt concentration and subjected to the compositional and structural analyses separately. Interestingly, the two sets of the immature 30S subunits are very distinct in both the protein composition and the 3D structure. The exposure to the high salt buffer does not destroy the known binding interdependences between the ribosomal proteins. But, it appears to have a destabilizing effect preferentially on the tertiary proteins in the 3′ domain. Structural data indicate that protein level of the 3′ domain is proportional to its rigidity, displaying an apparent coupling between the binding of proteins and the assembly of the rRNA secondary elements. Especially, the deficiency of S5 in structures from the high salt treated sample is correlated with a dramatic rotation of the head domain, as well as an alternative conformation of the 5′ end of the 16S rRNA, which suggests an underrecognized role of S5 in coordinating the 5′ end maturation and the 3′ domain assembly. Furthermore, our data reveal the possible location of the predicted helical stem formed by basepairing between the extra sequences in the 5′ and 3′ ends of the 17S rRNA. Overall, our results not only provide structural insights into the *in vivo* maturation of the 30S subunit, particularly at both early and late stages of the 3′ domain assembly, but also demonstrate the presence of multiple rRNA maturation checkpoints along the assembly pathway.

## Results

### Protein composition of the immature 30S subunits isolated with high salt buffer from the Δ*rbfA*Δ*rsgA* strain

Previously, we have determined protein levels of the immature 30S subunits isolated from the Δ*rbfA*Δ*rsgA* strain, using a relatively low salt concentration (150 mmol/L NH_4_Cl) in the sucrose cushion (Guo et al., 2013). Notably, a previous study showed that the salt concentration during purification could largely change the protein profile of isolated *in vivo* 50S assembly intermediates (Jiang et al., 2006). And it is known that the salt concentration is an important factor for reconstitutions of ribosomal subunits in *in vitro*. Typical *in vitro* 30S reconstitutions were carried out in a buffer containing 330 mmol/L KCl [for examples, see (Adilakshmi et al., 2008; Culver and Noller, 1999; Talkington et al., 2005)], and even higher salt concentrations were used in earlier studies (Traub and Nomura, 1968). Therefore, in order to interpret the compositional data based on isolated samples in a physiological context, it is necessary to compare the *in vivo* 30S intermediates obtained under different experimental conditions. It is conventional to use high salt buffers (for example, 300–1000 mmol/L NH_4_Cl) to remove bound factors or other undesired proteins to obtain active ribosomes or ribosomal subunits through centrifugation (Blaha et al., 2000; Rodnina and Wintermeyer, 1995; Selmer et al., 2006) or chromatography (Jelenc, 1980; Maguire et al., 2008) based purification techniques. In the present work, we decided to try a relatively harsh condition (1 mol/L NH_4_Cl) in the sucrose cushion to purify the immature 30S subunits from the Δ*rbfA*Δ*rsgA* strain. This would enable us to understand the effect of salt concentration on the subunit assembly and to compare the data acquired with different experimental conditions (Guo et al., 2013; Jomaa et al., 2011; Leong et al., 2013).

**Figure 1:**
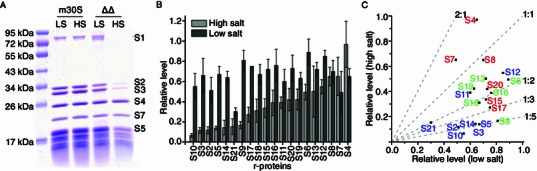
**Protein compositions of the*∆rbfA∆rsgA*immature 30S subunits isolated under different salt conditions**. (A) SDS-PAGE analysis of mature 30S (m30S) and *∆rbfA∆rsgA* (∆∆) immature 30S subunits isolated under low salt (LS) and high salt (HS) conditions. (B) QMS analysis of relative protein levels of the *∆rbfA∆rsgA* immature 30S subunits. (C) Comparison of relative levels of individual r-proteins under low salt and high salt conditions. The primary (1°), secondary (2°) and tertiary (3°) binding proteins are colored red, green and blue, respectively. The compositional data for the *∆rbfA∆rsgA* immature 30S subunits isolated under low salt condition are from a previous study (Guo et al., 2013)

For comparison, mature 30S subunits, which were derived from 70S ribosomes of the parental strain in a dissociating buffer containing 2 mmol/L Mg^2+^, were also prepared similarly with two different salt buffers (150 mmol/L vs 1 mol/L NH_4_Cl). As shown in Fig. 1A, based on the protein gel analysis, the mature 30S subunit tolerates the high salt exposure well; the protein levels of the mature 30S subunits that have undergone two different treatments are virtually identical. In contrast, immature 30S subunits show apparently reduced levels for some proteins, such as S1, S2, S3 and S5, when exposed to high salt buffer (Fig. 1A). To quantitatively analyze the protein composition of the immature 30S subunits isolated under high salt condition, we employed a previously established QMS method (Guo et al., 2013). Among all proteins, S1 was not included in the QMS analysis, as it dissociates readily from the 30S subunit during centrifugation-based purification. For a similar reason, although S21 was included in the QMS analysis, it is not discussed in the following sections.

As expected, high salt treatment has an obvious effect on most of the proteins (Fig. 1B), rendering an average occupancy of 0.36 (Table S3). Nevertheless, protein levels in this high salt treated sample still show a clear pattern, with the 3′ domain proteins mostly underrepresented. Particularly, the levels of proteins S10, S3, S2, S14, S5, S21 and S9 are severely reduced, less than 20% of those in the mature 30S subunit (Fig. 1B and Table S1). In fact, all of these proteins are tertiary or secondary proteins, and most of them (such as S10, S3, S2, S14 and S9) are localized in the 3′ domain. Also, in the high salt treatment sample, several primary binding proteins such as S17, S15 and S20 also show reduced levels to some extents (less than 50%) (Fig. 1B).

Therefore, taking into consideration the known binding interdependencies of the 30S proteins (Mizushima and Nomura, 1970), it can be concluded that the *in vivo* intermediates isolated with high salt buffers are a heterogeneous set of particles, and it may include intermediates that lack primary proteins in the 5′ and central domains. A major population of this set of *in vivo* particles isolated under high salt condition might still represent snapshots of 30S assembly intermediates trapped in early stages of the 3′ domain assembly, characterized by extremely low levels of secondary and tertiary proteins in the 3′ domain.

### High salt treatment preferentially weakens the binding of tertiary proteins in the head domain of the immature 30S subunits

Our QMS analysis on the high salt treated sample confirms that proteins in the immature 30S subunits are sensitive to salt concentrations in purification buffers. When the compositional data of the immature 30S subunits acquired under two salt conditions are plotted against each other, a recognizable pattern becomes apparent (Fig. 1C). The first observation is that high salt treatment decreases the occupancies of most of the proteins in the immature 30S subunits (Fig. 1C). The only exception is that two primary proteins S4 and S7 show relatively higher levels in the high salt treated sample. The second observation is that exposure to the high salt buffer affects the protein levels in a non-uniform manner. Previously, we reported that S21, S7 and S2 are the most underrepresented proteins (ranging from 0.3 to 0.5) in the low salt treated samples (Fig. 1B) (Guo et al., 2013). However, in the high salt treated sample, the most underrepresented one is S10, followed by S3, S2, S5, S14, S21 and S9 (lower than 0.20), clearly displaying a different pattern. In terms of the extent of protein level changes, the high salt treatment appears to have a more marked effect on tertiary proteins (Fig. 1C). In fact, all of the tertiary proteins except S12 and S11 have the largest changes in their relative levels, especially those in the 3′ domain (S10, S3, S2 and S14), as they are seen to have approximately a 5-fold change in occupancy. In addition, S9, a secondary binder in the 3′ domain, is also among the most affected group. The rest of secondary proteins are in the moderately affected group, with up to 2-fold change in occupancy. As to the primary proteins, they are the least affected, with slight changes of occupancy in both directions. Thus, taken together, the compositional difference in the two samples reveals that the high salt treatment preferentially facilitates dissociation of tertiary proteins in the 3′ head domain of the immature 30S subunits.

It is known that prior binding of late proteins in the 3′ domain could arrest the assembly process in certain stages, resulting in different kinetically trapped assembly intermediates (Mulder et al., 2010; Talkington et al., 2005). The immature 30S subunits isolated under low salt condition are low in S7 (Fig. 1B), and therefore they are likely a set of intermediates that are kinetically trapped due to slow binding of S7. The salt sensitivity of the 3′ domain tertiary proteins in the immature 30S subunits indicates that the premature association of these late proteins is probably non-native and relatively weak.

### Cryo-EM structures of the immature 30S subunits isolated under low salt condition from the Δ*rbfA*Δ*rsgA* strain

**Figure 2:**
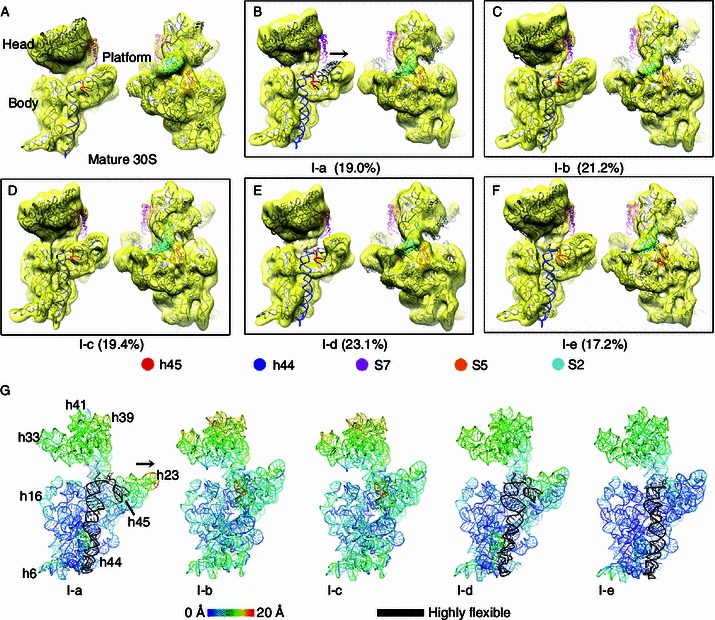
**Cryo-EM structures of low salt treated*∆rbfA∆rsgA*immature 30S subunits**. Density maps of the mature 30S (A) and low salt treated immature 30S subunits (B–F) are superimposed with the atomic model of the mature 30S subunit and displayed from both intersubunit and solvent views. h45, h44, S7, S5 and S2 are colored in red, blue, magenta, orange and cyan, respectively. Particle ratio of each structure is shown. (G) Distance deviations of the 16S rRNA in the five immature 30S structures from that of the mature 30S subunit are displayed as temperature maps, colored according to the scale bar. The highly flexible helices, such as h44 and h45 are colored black. Open conformation of the platform region in I-a is indicated by a black arrow (B and G)

Similar to our previous work, we applied cryo-EM single particle analysis to the low salt treated immature 30S particles from the Δ*rbfA*Δ*rsgA* strain. A multi-structure refinement method (Scheres, 2012) was used to probe structural intermediates of the immature 30S particles. On the 2D level, reference-free alignment and classification of all particles results in well resolved class averages, with fine details on both the head and body domains of the 30S subunits (Fig. S1A). With extensive tuning of the 3D classification parameters, the data were finally split into five classes, and the resulting five density maps are in a resolution of 13–18 Å. Similar to previous cryo-EM studies on the *in vivo* immature 30S subunits (Guo et al., 2013; Jomaa et al., 2011; Leong et al., 2013), no significant densities could be found beyond the 5′ and 3′ ends of the 16S rRNA, indicating that the terminal sequences of the 17S rRNA are flexible in these structures. However, these maps show a number of interesting differences (Movie S1). Firstly, S7 and S2 are seriously underrepresented in these structures (Fig. 2), which is highly consistent with the QMS-based compositional analysis (Fig. 1B). In fact, compared with the mature 30S map (Fig. 2A), none of structures has completely resolved S7. It is either totally (Fig. 2B) or partially (Fig. 2C–F) missing from the density maps. As to S2, it is relatively solid in two maps (Fig. 2C and 2F), but present in apparently sub-stoichiometric level in the rest three maps (Fig. 2B, 2D and 2E). Notably, S2 appears to have no strict binding dependence on S7, agreeing with our assumption that these structures represent intermediates that are arrested by prior binding of late proteins ahead of S7. Secondly, a very dramatic conformational difference lies at the 3′ minor domain of the 16S rRNA (h44 and h45). Structures of two classes (I-b and I-c), accounting for 40% of the immature 30S particles, show relatively rigid conformation for h44 and h45. In contrast, in the rest three maps (I-a, I-d and I-e), h44 is highly flexible, in completely undocked positions. Thirdly, the 30S platform region (h22–h24 of the central domain) appears to be in an open conformation in the I-a structure (Fig. 2B and 2G). This indicates that the late-stage assembly of the 30S subunit also involves the reorientation of the central domain of the 16S rRNA, consistent with a very recent X-ray footprinting data (Clatterbuck Soper et al., 2013). Lastly, although all the five structures have well resolved, rigid head domains, they appear in different rotational positions. This is most evident in the temperature maps of the 16S rRNA in these structures (Fig. 2G). Temperature maps were constructed by coloring the flexibly fitted atomic models of these structures according to their distance deviations from the structure of the mature 30S subunit. As shown, the head rotations in these maps are in a range of 10–18 Å, which is in sharp contrast to the immature 30S structures from the Δ*rimM* strain (Guo et al., 2013), with rotations in a much larger scale (up to 40 Å). Besides the large domain rotation, the temperature maps clearly show that the upper portion of h44 in the two structures with relatively ordered h44 (I-b and I-c, Fig. 2G) also deviates from the mature conformation, indicating that the decoding center in these immature 30S structures is not fully assembled.

In summary, in terms of protein composition and structural features, the immature 30S subunits obtained with low salt buffer is quite similar to the late-stage Group IV particles identified in a previous time-resolved structural study (Mulder et al., 2010), confirming that they are a set of late-stage intermediates that are close to completion.

### Cryo-EM structures of the immature 30S subunits isolated under high salt condition from the Δ*rbfA*Δ*rsgA* strain

**Figure 3:**
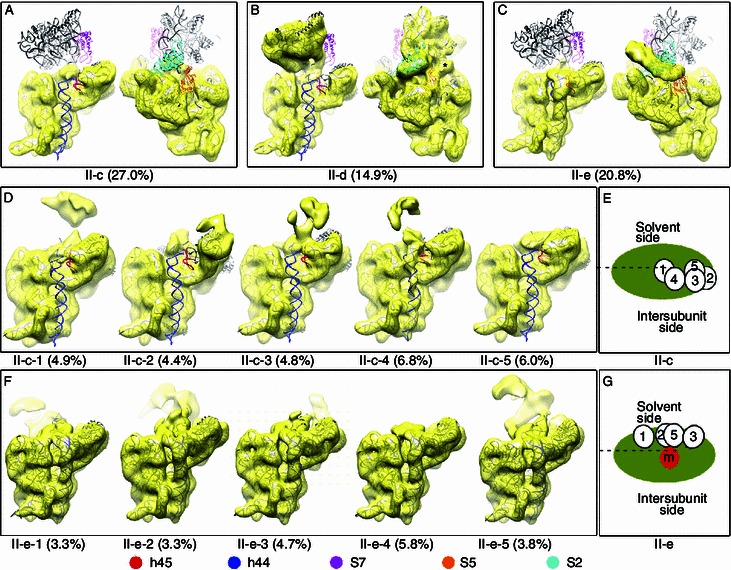
**Cryo-EM structures of high salt treated*∆rbfA∆rsgA*immature 30S subunits**. (A–C) Three representative density maps of high salt treated particles are superimposed with the atomic model of the mature 30S subunit and displayed from intersubunit and solvent views. (D) Subclass density maps of the II-c group are displayed from intersubunit view with the globally fitted atomic model of the mature 30S subunit (head domain excluded) superimposed. (F) Subclass density maps of the II-e group are displayed from intersubunit view with the flexibly fitted atomic model of the mature 30S subunit (head domain excluded) superimposed. The relative head domain orientations of the II-c and II-e subclass density maps are indicated as cartoon models in panel (E) and (G), respectively. In these models, the body domain is represented as a large green oval and the centers of the head domains as small white ovals with subclass number. The head domain position of the mature 30S subunit as a reference is shown in (G) as a red oval labeled with “m”. The head domain position of the II-e-4 map is not given in (G) since it has extremely low density in the head domain region

Similarly, we analyzed the immature 30S particles isolated under high salt condition using the identical image classification method. On the 2D level, class averages already show significant difference from the low salt treated particles, with a large portion of particles displaying extremely mobile head domains (Fig. S1B). On the 3D level, the particles were first separated into five classes. These five classes show very large structural differences (Figs. 3 and S2). Structures of the two classes (II-a and II-b) do not reveal any reliable structural details (Fig. S2), and are virtually biased global averages. The 2D reference-free classification of the particles from these two classes reveals that they lack the general defined shape of the 30S subunit (Fig. S2), suggesting they are a wide variety of heterogeneous particles, probably including low quality particles, aggregated particles, as well as very early-stage particles that are deficient in primary proteins in the 5′ and central domains. In contrast, the rest three classes (II-c, II-d and II-e) have well-resolved structural details (Fig. 3A–C), which indicates that they are truly populated structural intermediates. Among these three classes, the II-d structure is similar to structures from the low salt treated samples, with highly flexible h44 and h45 but a relatively rigid head domain (Fig. 3B). Interestingly, compared with the low salt structures, a small density blob appears at the mRNA entrance channel, which could be residual density for the 5′ leader sequence of the 17S rRNA (Figs. 3B and S3). The other two (II-c and II-e, Fig. 3A and 3C), in sharp contrast, display extremely mobile head domains, resulting in a cluster of smeared densities at the head domain location (Movies S2 and S3). There is also an apparent difference between the II-c and II-e structures: one is with a disordered 3′ minor domain (II-c, Fig. 3A), and the other with a well resolved 3′ minor domain (II-e, Fig. 3C). Based on the general structure features of the three class structures (Fig. 3A–C), we could group the high salt treated particles into two major populations: one with an extreme flexibility at the 3′ domain (II-c and II-e) and the other being typical late-stage particles (II-d) (Fig. S4).

The smeared densities at the head domain of two of these structures (II-c and II-e) suggest additional structural heterogeneity within these groups. Therefore, we further classified each of these two groups into five subclasses. In the subclass structures from the class II-c, different conformations of h44 are revealed (Fig. 3D). One of the five subclasses (II-c-4) shows partially resolved densities for h44, while the remaining four (20.1% particles in total) show completely undocked h44. Interestingly, in one of the subclass structure (II-c-2), the platform of the 30S subunit is again in an open conformation. Other than the conformational differences at the 3′ minor domain, the head domains of these subclass structures are clearly in different positions (Fig. 3E). Although the densities of head domains are relatively low compared with the body domain, recognizable shape of a typical head domain can be seen in lower threshold (Fig. S5 and Movie S2).

The subclass structures of the class II-e also reveal a number of interesting structural observations (Fig. 3F). Firstly, all five subclass structures have well-resolved h44 and h45. However, h44 in these structures is in different conformations, with its upper portion significantly bent, at varying extents, toward the 50S subunit direction, compared with the mature 30S structure (Fig. S6), indicating that the observed docking of h44 in these structures is probably premature. Secondly, the head domains in these maps are also at different positions (Fig. 3G, Fig. S5 and Movie S3). However, in terms of direction of the head rotation, subclass structures from the II-c and II-e groups are sharply different. While the head domain in the II-c subclass structures appears to swivel around the connecting hinge (h28) between the body domain and head domain (Fig. 3E and Movie S2), the head domain of the subclasses from the II-e group is almost bent 90 degree toward the back of the 30S subunit, and rotates in a totally different plane (Fig. 3G, Fig. S5 and Movie S3).

Therefore, our structural data on the high salt treated particles are consistent with the QMS data, indicating that many of the tertiary proteins in the 3′ domain are in fact absent from these intermediates. Consequently, loss of proteins at different rRNA locations greatly changes both the overall and local conformations of the 17S rRNA, particularly the 3′ head and minor domains.

## Discussion

### Effect of salt on the 30S subunit assembly

In the present work, we characterized the *in vivo* assembly intermediates from the Δ*rbfA*Δ*rsgA* strain isolated under two different salt conditions. Our results indicate that proteins in the *in vivo* intermediates are sensitive to salt treatment. While the exposure to 1 mol/L NH_4_^+^ largely changes both the protein profile and the rRNA conformation of the immature 30S particles, it has minimal effect on the mature 30S subunits. Especially, it is extremely effective in removing the 3′ domain tertiary proteins. This observation might have functional implications. Previous kinetic data show that the *in vitro* assembly of the 30S subunit often encounters kinetic traps in the 3′ domain, such as those caused by prior binding of late proteins, and predicts that assembly factors function to subvert these kinetic traps (Bunner et al., 2010; Mulder et al., 2010; Talkington et al., 2005). Our data further indicate that the premature associations of the 3′ domain proteins in these kinetically trapped intermediates are relatively weak, compared with those in the 5′ and central domains. This would mean that the requirement of non-physiologically high salt concentration in *in vitro* experiments is to minimize those relatively weak and non-productive associations of late proteins. In other words, the salt in the *in vitro* experiments partially compensates the functional role of assembly factors, by preventing the formation of kinetic traps. Indeed, a very interesting observation is that deletion of assembly factor genes confers salt resistance to *E. coli* cells (Hase et al., 2013; Hase et al., 2009), which also seems to suggest an equivalency between assembly factors and the salt ions.

The compositional differences in the immature 30S subunits obtained with different buffers also indicate that the QMS data based on purified samples might not truly reflect the situations inside the cell in every detail. One has to take into consideration the purification condition when it comes to the integration of data from different sources. It has drawn our attention that, in terms of individual protein levels, our previous QMS data of the immature 30S particles from the *rimM* null strain (Guo et al., 2013) show some discrepancies with another independently obtained QMS data from a similar strain (Leong et al., 2013). Besides the possible methodological errors introduced in mass spectrometry, the buffer systems (150 mmol/L vs 500 mmol/L NH_4_Cl in sucrose cushion) might be the major source of these differences (Table S3). Nevertheless, both our and their data are in general consistent with the current model of the ordered 5′ to 3′ assembly of the 30S subunit, showing that disruption of *rimM* causes the assembly arrest at the 3′ domain of the 16S rRNA (Guo et al., 2013).

The intracellular K^+^ concentration of the log phase *E. coli* cells is around 200 mmol/L (Schultz and Solomon, 1961), which is similar to our low salt condition (150 mmol/L NH_4_Cl). This indicates that our compositional data of the low salt treated samples (Guo et al., 2013) might largely retain the *in vivo* information. In terms of functional role of RbfA, our structural data on the low salt derived 30S particle from the Δ*rbfA*Δ*rsgA* strain is in consistent with recent findings that immature 30S subunits accumulated in different assembly factor-null strains share common structural features, such as the flexibility of the 3′ major and minor domains (Clatterbuck Soper et al., 2013; Guo et al., 2013; Jomaa et al., 2011; Leong et al., 2013). This indicates the presence of a common bottleneck during the late-stage assembly, and suggests that factors contribute not only to a single assembly event but to a global structural reorganization, probably in a concerted manner (Clatterbuck Soper et al., 2013; Leong et al., 2013).

As to the high salt derived particles, it appears that they might not have a direct functional relevance to RbfA and RsgA, due to the high salt treatment. However, our compositional data show that the high salt treatment does not destroy the relationship in the Nomura map, and the relative levels of the primary, secondary and tertiary proteins in these particles are exactly in a descending order (Fig. 1C). Furthermore, our structural classification successfully distinguishes the populations of the high salt washed particles in different conformational groups (Fig. 3). Consistently, both the protein composition and structural features of the two major groups correlate well with the previously reported *in vitro* assembly intermediates, corresponding to the Group II and IV particles, respectively (Mulder et al., 2010), suggesting that these structures might virtually reflect intermediates at different temporal stages. Altogether, our results indicate that the high salt treatment has largely changed the structures of the *in vivo* intermediates, and likely returned some of the late-stage particles to the entry stage of the 3′ domain assembly. Therefore, by varying the salt concentration in the sucrose cushion, we might have captured the 30S assembly intermediates at two distinctive stages.

### Possible location of the precursor sequences in the 5′ and 3′ ends of the 17S rRNA

**Figure 4:**
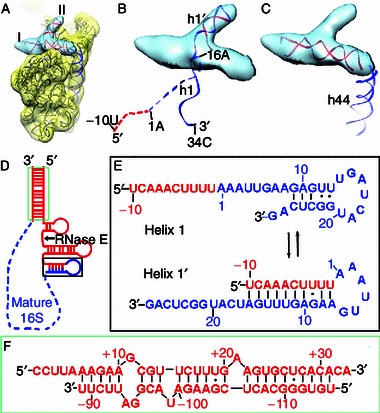
**Visualization of the extra 5′ and 3′ ends of the 17S rRNA**. (A) The density map of the II-e-3 subclass is displayed in transparent yellow, with flexibly fitted atomic model superimposed. A “T” shaped extra piece of densities (composed of two rods, I and II) is colored light blue. (B) Compatibility of the rod II with the alternative conformation of the 5′ end helix (h1′). The mature and precursor sequences are colored blue and red, respectively. Residues −10 to −1 of the leader sequences and 1–16 of the mature 16S rRNA in its native conformation are shown as dashed line. As indicated, the 5′ leader sequences together with the mature sequences (residues 1–16) could flip almost 180 degree and fold into an alternative structure (h1′). Also see Movie S4. (C) Compatibility of the rod I with the predicted helical stem formed between the precursor sequences of the 5′ and 3′ ends of the 17S rRNA. A segment of h44 in the mature 30S structure is also shown, indicating the continuity between the rod I and h44. For illustration, a standard helix of ~27 bp is fitted in the map. (D) Secondary structure prediction of the complete 17S rRNA. Sequences of the mature 16S rRNA are indicated as blue dashed line and the precursor sequences are colored red. The prediction was performed with a standalone version of RNA Structure (Version 4.6) using free energy minimization (Mathews et al., 2004). (E) Competing conformations of the 5′ end of the 16S rRNA, as two equilibrating states (h1 and h1′). The alternative h1′ is formed by basepairing between residues 7–16 and −1 to −10 of the 5′ leader sequences. (F) Predicted helical stem formed between the unprocessed 5′ and 3′ precursor sequences at the termini of the 17S rRNA

A very important observation from the structures of high salt treated samples is that in three subclass structures of the II-e group (II-e-2, II-e-3 and II-e-5), we found a piece of well resolved additional mass, located on the shoulder region of the 30S subunit (Fig. 4A). This mass is like a deformed letter “T”, composed of a long rod with an orthogonal short rod (Fig. 4A). The two rods (I and II) of this T-shaped density cluster match well with the diameter of an rRNA helix, suggesting they are from a long helix (I) and a relatively vertical short helix (II). Careful examination of this additional mass reveals that it is not from the known fragments of the 16S rRNA (Movie S4). In terms of the density continuity, the longer rod clearly connects to the h44-h45 (Fig. 4C), while the shorter one is near the location of h1 in the mature 30S structure (Fig. 4B). Previously, we showed that a majority of the rRNA species in the immature 30S particles from the Δ*rbfA*Δ*rsgA* strain is unprocessed at both ends (Guo et al., 2013), being the cleavage product of RNase III. Therefore, it is highly likely this extra mass is from the precursor sequences of the 17S rRNA.

Previous genetic and biochemical data have established a helical switching model for the maturation of the 5′ end of the 17S rRNA (Dammel and Noller, 1993; Young and Steitz, 1978). In this model, the 5′ mature sequence of the 17S rRNA would form a different secondary structure (h1′) other than that seen in the mature 16S form (h1) (Fig. 4E), with base paring between residues 7 to 16 and −1 to −10 of the leader sequence (Young and Steitz, 1978). Disturbing the transition from h1′ to the mature h1, such as mutations on protein S5 (G28D) (Roy-Chaudhuri et al., 2010) or on the 16S rRNA (C23U) (Dammel and Noller, 1993) would introduce defects in the 17S rRNA processing and the 30S subunit assembly. Additionally, overexpression of RbfA, which binds to the neck of the 30S subunit, at a helical junction of h28, h27, h18 and h1 (Datta et al., 2007), could suppress the assembly defects in the C23U rRNA mutant strain (Dammel and Noller, 1995), which is in further support of this helical switching model for the maturation of the 17S rRNA 5′ end. As to the maturation of the 3′ precursor sequences, based on the secondary structure prediction, multiple rRNA duplexes could potentially form from the extra nucleotides of the 17S rRNA (115 nucleotides at the 5′ end and 33 nucleotides at the 3′ end) (Fig. 4D). Especially, the 5′ and 3′ ends of the 17S rRNA have complementary sequences (Fig. 4F), and could form a long helix (Young and Steitz, 1978).

The shorter rod of this extra mass could exactly accommodate a helical stem of ~10 base pairs (Fig. 4B and Movie S4), matching well with the predicted alternative helix (h1′) in the 5′ end of the 17S rRNA (Dammel and Noller, 1993; Roy-Chaudhuri et al., 2010; Young and Steitz, 1978). The longer rod of this extra mass is roughly equivalent to an rRNA helix of 26 base pairs (Fig. 4C). Since this longer rod is close to the very 3′ end of the 16S rRNA, it is likely the rRNA duplex formed between the two unprocessed ends of the 17S rRNA (Movie S4). Therefore, we might directly visualize an earlier conformation of the 17S rRNA that is with base paired precursor sequences from the 5′ and 3′ ends. Although it is not possible to further characterize structural details of this extra mass, our data from the high salt treated sample reveals, at least partially, the location of the precursor sequences of the 17S rRNA, confirming the presence of complementary base pairing within the 5′ end, as well as between the 5′ and 3′ ends in the 17S rRNA precursor.

### S5 couples the 5′ end maturation of the 17S rRNA with the 3′ domain assembly of the 30S subunit

**Figure 5:**
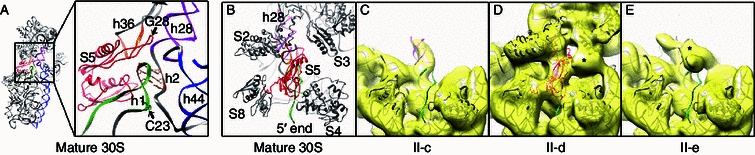
**Role of S5 in coupling the 17S rRNA 5′ end maturation with the assembly of the 30S 3′ domain**. (A) A zoom-in view of S5 and its surrounding components in the mature 30S structure. S5, h1, h2, h28, h36 and h44 are colored red, forest green, sienna, magenta, orange and blue, respectively. Residue G28 in S5 and C23 in the 16S rRNA, which are important for the 5′ end maturation of the 17S rRNA are indicated by arrows. C23 and nucleotides forming a pseudoknot h2 are highlighted with side chains. (B) Close-up solvent view of the atomic model of the mature 30S subunit, showing S5 and its neighbors, such as S2, S3, S4, S8 and h1, with neighboring ribosomal proteins colored black and h1 in forest green. (C–E) Density maps of II-c (C), II-d (D) and II-e (E), superimposed with atomic model of the mature 30S subunit, are displayed from the same view as (B). For clarification, components of the head domain of the atomic model and S5 are removed in (C) and (E) since there are only very limited densities at the head domain location in these maps. As shown in (C–E), the presence of S5 is coupled with the rigidity of the head domain, as well as with the mature h1 conformation. The asterisks in (D and E) denote the residual densities of the 5′ leader sequences in the 17S rRNA

S5 is a tertiary protein that binds at a helical junction (Culver et al., 1999), formed by h1, h2, h28, h36 and h44 (Fig. 5A), including rRNA segments from all four domains of the 16S rRNA. Previous work has implicated an important role of S5 in translation, showing that several *ram* mutations could be mapped to S5 (Ito and Wittmann, 1973; Kirthi et al., 2006; Piepersberg et al., 1975). Recently, in a series of studies it was shown that the miscoding phenotype of one S5 *ram* mutation (G28D), as well as a few other mutant strains (Δ*rng,* Δ*rpsO,* Δ*rimM* and Δ*ksgA*), is a consequence of cellular defects in ribosome assembly, particularly the faulty maturation of the 5′ end of the 17S rRNA (Kirthi et al., 2006; Roy-Chaudhuri et al., 2010; Roy-Chaudhuri et al., 2008), which have demonstrated a functional link between the decreased ribosome fidelity and the maturation defect of the 5′ end of the 17S rRNA. The site of this specific S5 mutation (G28D) is located in a protruding loop inserted into the interface of several rRNA helices, including h28, h36, h44 and h1 (Fig. 5A), manifesting an essential role of S5 in the conformational maturation of the 17S rRNA.

Very interestingly, we found that the dramatic head rotation observed in the high salt treated samples (II-c and II-e) is well correlated with the absence of S5 in these structures (Fig. 3A and 3C, Fig. 5, Movies S2 and S3). Specifically, we observed a correspondence between the local structural difference at the S5-h1-h28 interface and global conformational difference of the 17S rRNA (Fig. 5). In all structures from the low salt treated sample, S5 is well represented (Fig. 2B–F and Fig. S3), and the head domain in these structures is relatively rigid, although in slightly different rotational position (~10–15 Å in scale). The 5′ mature sequence of the 17S rRNA in these structures also appears to be in a mature conformation, but the leader sequence at the 5′ end is highly flexible (Fig. S3). In contrast, only one structure of the high salt treated sample displays well resolved densities for S5, and the 5′ mature sequence in the 17S rRNA and the head domain are again relatively rigid in this structure (Figs. 5D and S3). Other structures from the high salt treated sample are clearly depleted in S5 (Figs. 3 and 4), and they display very dramatic motions at the head domain and the 3′ minor domain. Detailed analysis indicates that local conformational differences on h28 and h1 might account for structural dynamics of the head domain. As shown, in the two S5-deficient structures with extremely mobile head domain, h28 is not well resolved (Fig. 5C and 5E). Other than that, in these S5-deficient structures, the very 5′ end of the 16S rRNA (single strand region, residues 1–8), as well as the first helix h1, is also highly flexible (Fig. 5C and 5E), indicating that the 5′ end of the 16S rRNA in these structures is in a conformation distinctive from its mature state. This also means that the central pseudoknot h2, formed by basepairing between one extension strand of h28 and the terminal loop of h1 (Fig. 5A), is not formed in these structures.

Altogether, these data suggest the binding of S5 facilitate the transition from h1′ to h1 and the stabilization of the native S5-h1-h28 interface. Consequently, the 3′ head domain acquires rigidity from a stabilized h28 and commits to further assembly. Thus, S5 appears to be an important player at the relatively early stage (probably the entry stage) of the 3′ domain assembly. An integrated role of S5 is to couple the maturation of the 5′ end of the 17S rRNA with the assembly of the 3′ domain, through the modulation of the S5-h1-h28 interface. Notably, this view is consistent with previous kinetic and time resolved structural data showing that S5 binds prior to most of the 3′ domain proteins, roughly at the entry stage of the 3′ domain assembly (Chen and Williamson, 2013; Mulder et al., 2010).

### Quality control of the 30S assembly

It is generally believed that the maturation of the 17S rRNA would complete before the 30S subunit is incorporated into the 70S ribosomes. Recent biochemical and structural data from ours and others are consistent with this view, showing that the major rRNA species in the immature 30S particles from factor-null strains are unprocessed at both the 5′ and 3′ ends (Clatterbuck Soper et al., 2013; Davies et al., 2010; Goto et al., 2011; Guo et al., 2013; Jomaa et al., 2011; Leong et al., 2013), and especially these immature 30S particles are highly dynamic at the 3′ minor domain (h44) [(Boehringer et al., 2012; Guo et al., 2013; Jomaa et al., 2011; Leong et al., 2013) (Clatterbuck Soper et al., 2013) and current study]. The 3′ minor domain hosts a number of intersubunit contact sites that are essential for the 50S association, which explains why substantial levels of immature 30S particles accumulate in such a variety of mutant strains. This suggests that the rate-limiting docking of h44, which is also observed on the mature 16S rRNA in *in vitro* reconstitution experiments (Adilakshmi et al., 2008; Dutca and Culver, 2008), could provide an intrinsic quality control on subunit production.

However, there are also lines of evidence showing that the immature 30S subunits containing 17S rRNA precursor are also capable of being incorporated into 70S ribosomes and polysomes (Clatterbuck Soper et al., 2013; Davies et al., 2010; Li et al., 1999; Mangiarotti et al., 1974; Roy-Chaudhuri et al., 2010), and an alternative idea is that the final maturation of the 17S rRNA takes place on the 70S or polysomes (Mangiarotti et al., 1974). Although the exact processing status of the 17S rRNA contained in these 70S ribosomes is not conclusively analyzed in each of these cases, some of them appear to be completely unprocessed at both ends (Davies et al., 2010; Li et al., 1999). Nevertheless, the above two models might not be exclusive, because the majority of the 17S rRNA is still in the free 30S fractions. Substantial incorporation of immature 30S subunit into the 70S ribosome itself could be a cellular disorder due to the processing defect in the rRNA maturation. Indeed, characterization of some types of these 17S rRNA-containing ribosomes reveals a defect in translational fidelity (Davies et al., 2010; Roy-Chaudhuri et al., 2010), and more importantly, it was recently demonstrated that the immature 30S subunits in these 70S ribosomes could trigger degradation of defective 70S ribosomes by YbeY and RNase R (Jacob et al., 2013). Therefore, these results show that bacterial cells possess multiple quality control systems that make use of the rRNA maturation at different stages to ensure the integrity of the 70S ribosome.

## Materials and methods

### Isolation of the immature and mature 30S subunits

Mature 30S subunits were purified from *E. coli* A19 strain (Hfr, *rna-19*, *gdhA2*, *his-95*, *relA1*, *spoT1*, *metB1*), and immature 30S subunits from an A19 derivative strain (*∆rsgA∆rbfA*) (Guo et al., 2013). Both the mature and immature 30S subunits were prepared under two salt conditions, with a sucrose cushion containing 150 mmol/L (low salt) or 1 mol/L NH_4_Cl (high salt). The low salt purification procedures were previously described (Guo et al., 2013). The high salt purification is similar with modifications. Specifically, cells (A19, A19-*∆rsgA∆rbfA*) were cultured in LB medium at 37°C to reach log phase and harvested by centrifugation in a JLA 10.500 rotor (Beckman Coulter) at 5,500 rpm for 20 min. Cell pellets were resuspended in a lysis buffer [10 mmol/L Tris-HCl (pH 7.8), 10 mmol/L MgCl_2_, 60 mmol/L NH_4_Cl, 0.5 mmol/L CaCl_2_, 0.1 mmol/L ethylene-diamine tetraacetic acid (EDTA), 1 mmol/L dithiothreitol (DTT)] and ground with 0.5 mm glass beads (Scientific Industries) for 30 min at 4°C. Cell debris was removed by centrifugation in a JA 25.50 rotor (Beckman Coulter) at 15,500 rpm for 1 h, and the supernatant was gently transferred onto the top of 5 mL sucrose cushion [30% sucrose, 10 mmol/L Tris-HCl (pH 7.8), 10 mmol/L MgCl_2_, 0.1 mmol/L EDTA, 1 mmol/L DTT, 1 mol/L NH_4_Cl] and centrifuged in a 70 Ti rotor (Beckman Coulter) for 18 h at 28,000 rpm. The pellet after centrifugation was then dissolved in a gradient buffer [10 mmol/L Tris-HCl (pH 7.8), 10 mmol/L MgCl_2_, 60 mmol/L NH_4_Cl, 0.1 mmol/L EDTA, 1 mmol/L DTT], layered on the top of 10%–40% sucrose gradient in the same buffer, and centrifuged in a SW32 rotor (Beckman Coulter) at 30,000 rpm for 7 h. The gradients were initially prepared using the Gradient Master 108 (BioComp), and analyzed with A254 absorbance using a Teledyne ISCO fractionation system. The resulting 30S and 70S peaks were collected. The 70S ribosomes from the parental strain (A19) were separated into 30S and 50S subunits by changing the buffer into the dissociation buffer (same as gradient buffer, except 2 mmol/L MgCl_2_). Mature 30S subunits were then obtained through centrifugation with a 10%–40% gradient (2 mmol/L MgCl_2_).

### Quantitative mass spectrometry

In order to quantitatively determine abundance of ribosomal proteins in the high salt treated immature 30S subunits, a TMT-based (tandem mass tags) (Thompson et al., 2003) quantitative mass spectrometry (QMS) was applied, as previously described (Guo et al., 2013). Briefly, equal amounts of samples (exactly the same A260 units) were separated on a 15% SDS-PAGE gel. The protein bands except S1 were excised from the gel, reduced with 10 mmol/L of DTT and alkylated with 55 mmol/L iodoacetamide. After the overnight in-gel digestion by trypsin (Promega), the peptides were recovered by 1% TFA (trifluoroacetic acid) solution. Peptides were then labeled with TMT reagents (Thermo, Pierce Biotechnology) according to the manufacturer’s instruction (TMT 130 and 131 for the immature and mature 30S subunits, respectively). For LC-MS/MS analysis, the TMT-labeled peptides were separated by a 60-min gradient elution at a flow rate of 0.250 μL/min with an EASY-nLCII™ integrated nano-HPLC system (Proxeon), which is directly interfaced with a Q Exactive mass spectrometer (Thermo Scientific). The analytical column was a fused silica capillary column (75 μm ID, 150 mm length; packed with C-18 resin). Mobile phase A consisted of 0.1% formic acid and mobile phase B consisted of 100% acetonitrile and 0.1% formic acid. The Q Exactive mass spectrometer was operated in the data-dependent acquisition mode using the Xcalibur 2.1.3 software and there was a single full-scan mass spectrum in the Orbitrap (400–1800 m/z, 30,000 resolution) followed by 10 MS/MS scans in the quadrupole collision cell using the higher energy collision dissociation. The MS/MS spectra from each LC-MS/MS run were searched against the selected database using Proteome Discovery searching engine. The searching parameters are listed below: peptides ms tolerance of 20 ppm; ms/ms tolerance of 20 mmu; carbamidomethylation of Cys, TMT of lysine and peptide N terminal as the fixed modification, oxidation on Met as the variable modification. Peptides with high confidence were used for protein identification and MS/MS spectra for all matched peptides were manually interpreted and confirmed. Ratios of 130:131 for each of the ribosomal proteins were calculated with outliers removed by Grubbs’ test (http://graphpad.com/quickcalcs/Grubbs1.cfm). Only ratios with two or more tryptic peptides from the same protein were used to calculate the means and the standard deviations. The statistics of the TMT-based QMS measurements were summarized in Table S1.

### Cryo-sample preparation and data collection

Quantifoil 2/4 grids (Quantifoil Micro Tools GmbH) were coated with a thin layer of continuous carbon (K950X, EMITech) and glow-discharged in a Harrick Plasma Cleaner (PDC-32G) for 30 s. Grids were prepared with an FEI Vitrobot Mark IV at 4°C as previously described (Guo et al., 2011). The final concentration for blotting was ~60 nmol/L. Cryo-grids were examined in an FEI Titan Krios, and the data were collected under low-dose conditions (~20 e^−^/Å^2^) on an FEI Eagle 4 k × 4 k CCD using AutoEMation (Lei and Frank, 2005). The images of the low salt and high salt treated particles were collected at a nominal magnification of 59,000×, but with different acceleration voltages, at 300 KV and 120 KV, respectively.

### Image processing

4365 and 5590 micrographs were obtained for low salt and high salt treated immature 30S samples, respectively. After screening for ice contamination and astigmatism, 3238 and 4210 micrographs, for the low salt and high salt treated samples, respectively, were kept for further processing. All the micrographs were decimated by a factor of two, resulting in 3 Å and 2.62 Å in effective pixel size, for low salt and high salt intermediates, respectively. Contrast transfer function (CTF) parameters estimation and particle picking were done using SPIDER software package (Shaikh et al., 2008a). Raw particles were windowed using a local cross-correlation function based method (Rath and Frank, 2004). Automatically picked particles were manually verified using a correspondence analysis based method (Shaikh et al., 2008b). Finally, 219,169 and 160,264 particles, for low salt and high salt intermediates, respectively, were kept and subjected to single particle analysis. To explore the structural heterogeneity, classification was applied both at two dimensional (2D) and three dimensional (3D) levels, using RELION, an empirical Bayesian approach (Scheres, 2012). Both samples were first classified into 200 classes at 2D level in 25 iterations with an angle step of 5 degree, and bad particles were excluded from further analysis. At the 3D level, different parameters and class numbers have been tested. The initial model in 3D classification was generated by low-pass filtering (40 Å) of a previous cryo-EM map of the mature 30S subunit (Guo et al., 2011). The particles were finally split into five groups in fifty iterations until the classification was stabilized. To further determine head orientation of the class II-c and II-e particles, these particles were subjected to additional rounds of 3D classification using RELION, resulting in five subclasses for each group. Each subclass was further refined using SPIDER according to standard reference projection matching procedures (Shaikh et al., 2008a). The final resolutions of the refined structures were estimated using FSC (Fourier shell correlation) 0.5 cutoff criterion (Fig. S7). The statistics of image processing and the general features of the density maps were summarized in Table S2.

### Flexible fitting and map analysis

A crystal structure of the 30S subunit (PDB ID: 3OFA) (Dunkle et al., 2010) was used as the initial model. For structures derived from low salt treated sample, the crystal structure of the 30S subunit was docked in each map and subjected to a flexible fitting method based on molecular dynamics simulation **(**MDFF) (Trabuco et al., 2008), using NAMD (Phillips et al., 2005). The simulations were performed with molecules placed in vacuum, monitored by the global correlation coefficients between the density map and the simulated model. For class II-e and its subclasses (II-e-1, II-e-2, II-e-3, II-e-4 and II-e-5), due to the low densities at the 3′ head domain of the 30S subunit, only coordinates of the body domain (including S4, S6, S8, S11, S12, S15, S16, S17, S18, S20 and S21, nucleotides 2–940 and 1375–1501 of the 16S rRNA) were subjected to the MDFF. After fitting, the coordinates of the rRNA in all simulated atomic models were extracted and aligned to that of the mature 30S subunit, using the nucleotides 2–900 as the reference for alignment. Temperature maps were generated in PyMOL (Schrodinger, 2010) through calculating the deviation of the rRNA coordinates in each fitted model from that of the mature 16S rRNA. The scripts for temperature map building are accessible at http://pldserver1.biochem.queensu.ca/rlc/work/pymol/. PyMOL and Chimera (Pettersen et al., 2004) were used for structural analysis and figure preparation.

## Accession Codes

The cryo-EM maps have been deposited in the EMDataBank (EMDB codes 5900, 5904, 5905, 5906, 5907, 5908, 5909 and 5910, for the I-a, I-b, I-c, I-d, I-e, II-c-3, II-d and II-e-3 structures, respectively).

## Electronic supplementary material

Below is the link to the electronic supplementary material.

Supplementary material 1 (PDF 650 kb)

Supplementary material 2 (MP4 8120 kb)

Supplementary material 3 (MP4 8038 kb)

Supplementary material 4 (MP4 6645 kb)

Supplementary material 5 (MP4 6392 kb)
