# Supplementary material for: Structural insights into the assembly of the 30S ribosomal subunit in vivo: functional role of S5 and location of the 17S rRNA precursor sequence
Source: Protein Cell. 2014 Mar 28;5(5):394–407. doi: 10.1007/s13238-014-0044-1 (PMC3996153; doi:10.1007/s13238-014-0044-1)
Supplement: Supplementary file 1 — Supplementary material 1 (PDF 650 kb) [file 13238_2014_44_MOESM1_ESM.pdf]

Supplemental Materials

Structural insights into the assembly of the 30S ribosomal subunit *in vivo*: functional role of S5 and location of the 17S rRNA precursor sequence

Zhixiu Yang[1,3], Qiang Guo[1,3], Simon Goto[2], Yuling Chen[1], Ningning Li[1], Kaige Yan[1], Yixiao Zhang[1], Akira Muto[2], Haiteng Deng[1], Hyouta Himeno[2], Jianlin Lei[1,*], Ning Gao[1,*]

[1]Ministry of Education Key Laboratory of Protein Sciences, Center for Structural Biology, School of Life Sciences, Tsinghua University, Beijing 100084, China

[2]Department of Biochemistry and Molecular Biology, Faculty of Agriculture and Life Science, Hirosaki University, Hirosaki 036-8561, Japan

[3]These authors contributed equally to this work

*For correspondence: ninggao@tsinghua.edu.cn, or jllei@tsinghua.edu.cn

List of Supplemental Data

Supplemental Table (3)

Supplemental Figures (6)

Supplemental References

Supplemental Table S1 QMS analysis of the immature 30S subunits from the Δ*rbfA*Δ*rsgA* strain (high salt condition)

r-proteins	ΔΔ/mature	Standard Deviation	Coverage (%)	Unique Peptides	Peptide Spectrum Matches (PSMs)
S10	0.07	0.02	69.90	11	157
S3	0.12	0.03	85.41	24	327
S2	0.12	0.04	84.23	20	352
S5	0.14	0.02	84.43	14	315
S14	0.14	0.07	58.42	11	69
S21	0.15	0.08	54.93	9	47
S9	0.17	0.04	66.92	13	81
S17	0.27	0.07	76.19	9	50
S18	0.31	0.11	57.33	8	127
S15	0.34	0.09	70.79	9	56
S16	0.39	0.13	73.17	10	80
S11	0.39	0.06	61.24	9	117
S20	0.42	0.11	58.62	9	72
S19	0.42	0.10	76.09	9	128
S6	0.49	0.08	92.37	14	207
S13	0.50	0.25	77.97	15	259
S12	0.55	0.07	64.52	12	92
S8	0.65	0.09	78.46	17	235
S7	0.65	0.19	84.92	27	383
S4	0.97	0.23	78.64	26	469

Supplemental Table S2 Summary of Cryo-EM image processing and map features

Salt condition	Total particle number	Group name		State	Particle number	Ratio (%)	Resolution (Å)	Description
Low salt	219,169	I-a		late	41,709	19.0	15.2	S2 and S7 are severely underrepresented, h44 and h45 are highly flexible, and the platform region is in an open conformation.
		I-b		late	46,414	21.2	13.5	S7 is moderately underrepresented, while S2 is not. And h44 and h45 are in rigid conformations.
		I-c		late	42,521	19.4	14.4	Similar to I-b with a lower S2 occupancy.
		I-d		late	50,721	23.1	17.8	S2 and S7 are moderately underrepresented; h44 and h45 are highly flexible.
		I-e		late	37,804	17.2	14.3	Similar to I-b but with highly flexible h44.
High salt	160,264	II-a		mixture	35,644	22.2	24.2	With no reliable structural details.
		II-b		mixture	24,012	15.0	28.4	Similar to II-a.
		II-c		intermediate	43,308	27.0	15.3	The head domain, h44 and h45 are highly flexible. S5 is absent. Also, h1 is not in its native conformation.
		Subclass	II-c-1	intermediate	7,833	4.9	21.8	Similar to II-c, density for the head domain is visible only at a very low threshold.
			II-c-2	intermediate	7,082	4.4	21.0	Similar to II-c, head domain seems to extend toward the platform.
			II-c-3	intermediate	7,766	4.8	21.2	Similar to II-c.
			II-c-4	intermediate	10,945	6.8	18.8	Similar to II-c, but h44 is visible when a lower threshold is used.
			II-c-5	intermediate	9,682	6.0	19.1	Similar to II-c-1.
		II-d		late	23,956	14.9	15.2	S7 is seriously underrepresented but with relatively higher occupancy compared with low salt structures. The h44 and h45 are

							highly flexible. A small density blob appears to be at the mRNA entrance channel
		II-e		intermediate	33,344	20.8	14.9	S5 is absent and helix 1 is not in its native conformation. The head domain is partially visible and has a ~90° rotation toward solvent side. The h44 and h45 are well resolved. However, the upper part of helix 44 is distorted toward intersubunit direction.
		Subclass	II-e-1	intermediate	5,258	3.3	19.5	Similar to II-e
			II-e-2	intermediate	5,243	3.3	20.6	Similar to II-e-1, but with a different head orientation. Also a discernible “T” shaped extra density mass is seen on the shoulder.
			II-e-3	intermediate	7,536	4.7	18.3	Similar to II-e-2, with a different head orientation.
			II-e-4	intermediate	9,281	5.8	18.3	The body domain is similar to all the other subgroups of II-e but the head domain is completely missing.
			II-e-5	intermediate	6,026	3.8	19.7	Similar to II-e-2, with a slightly different head orientation.

Supplemental Table S3 Summary of the different QMS data on *in vivo* immature 30S subunits and their differences in purification buffers.

Strain	Mean occupancy	Buffer	Reference
Δ*rim M*	0.53	**lysis buffer:** 20 mM Tris-HCl (pH 7.5), 150 mM NH_4Cl, 10 mM $Mg(OAc)_2$ and 0.5 mM ethylene diamine tetra-acetic acid (EDTA)<br>**cushion**: 20 mM Tris–HCl (pH 7.5),150 mM NH_4Cl, 10 mM $Mg(OAc)_2$, 0.5 mM EDTA and 1.1 M sucrose<br>**gradient buffer:** 20 mM Tris-HCl (pH 7.5), 150 mM NH_4Cl, 10 mM $Mg(OAc)_2$, 10%-40% sucrose<br>**store buffer**: same as gradient buffer (without sucrose)	(Guo et al., 2013)
Δ*rim M*	0.75 (estimated)	**lysis buffer:** 20 mM Tris-HCl (pH 7.5), 10 mM $Mg(OAc)_2$, 100 mM NH_4Cl, 0.5 mM EDTA, and 3 mM 2-mercaptoethanol<br>**cushion:** 20 mM Tris-HCl (pH 7.5), 10.5 mM $Mg(OAc)_2$, 500 mM NH_4Cl, 0.5 mM EDTA, 3 mM 2-mercaptoethanol<br>**resuspension buffer**: 10 mM Tris-HCl (pH 7.5), 10.5 mM $Mg(OAc)_2$, 100 mM NH_4Cl, 0.5 mM EDTA, and 7 mM 2-mercaptoethanol<br>**gradient buffer**: 10 mM Tris-HCl (pH 7.5), 10 mM $Mg(OAc)_2$, 60 mM NH_4Cl, and 3 mM 2-mercaptoethanol, 10%-30% sucrose<br>**store buffer:** same as gradient buffer (without sucrose)	(Leong et al., 2013)
Δ*rbfA*Δ*rsgA*	0.67	**lysis buffer**: 20 mM Tris-HCl (pH 7.5), 150 mM NH_4Cl, 10 mM $Mg(OAc)_2$ and 0.5 mM EDTA<br>**cushion:** 20 mM Tris-HCl (pH 7.5), 150 mM NH_4Cl, 10 mM $Mg(OAc)_2$, 0.5 mM EDTA and 1.1 M sucrose<br>**gradient buffe**r: 20 mM Tris-HCl (pH 7.5), 150 mM NH_4Cl, 10 mM $Mg(OAc)_2$, 10%-40% gradient<br>**store buffe**r: same as gradient buffer (without sucrose)	(Guo et al., 2013)
Δ*rbfA*Δ*rsgA*	0.36	**lysis buffer:** 10 mM Tris-HCl (pH 7.8), 10 mM $MgCl_2$, 60 mM NH_4Cl, 0.5 mM $CaCl_2$, 0.1 mM EDTA, 1 mM DTT	present data

		**cushion**:10 mM Tris-HCl (pH 7.8), 10 mM $MgCl_2$, 0.1 mM EDTA, 1 mM DTT, 1 M NH_4Cl, 30% sucrose<br>**gradient buffer**: 10 mM Tris-HCl (pH 7.8), 10 mM $MgCl_2$, 60 mM NH_4Cl, 0.1 mM EDTA, 1 mM DTT<br>**store buffer**: same as gradient buffer (without sucrose)	
Δ*rsgA*	0.71	**lysis buffer:** 20 mM Tris-HCl (pH 7.5), 10.5 mM $Mg(OAc)_2$, 100 mM NH_4Cl, 0.5 mM EDTA, and 3 mM 2-mercaptoethanol<br>**cushion**: 20 mM Tris-HCl (pH 7.5), 10.5 mM $Mg(OAc)_2$, 500 mM NH_4Cl, 0.5 mM EDTA, and 3 mM 2-mercaptoethanol, 1.1 M sucrose<br>**resuspension buffer**: 10 mM Tris-HCl (pH 7.5), 10.5 mM $Mg(OAc)_2$, 100 mM NH_4Cl, 0.5 mM EDTA, and 7 mM 2-mercaptoethanol<br>**gradient buffer**: 10 mM Tris-HCl (pH 7.5), 10 mM $Mg(OAc)_2$, 60 mM NH_4Cl, 3 mM 2-mercaptoethanol,10%-30% gradient<br>**store buffer**: 10 mM Tris-HCl (pH 7.5), 10 mM $Mg(OAc)_2$, 60 mM NH_4Cl, 3 mM 2-mercaptoethanol	(Jomaa et al., 2011)

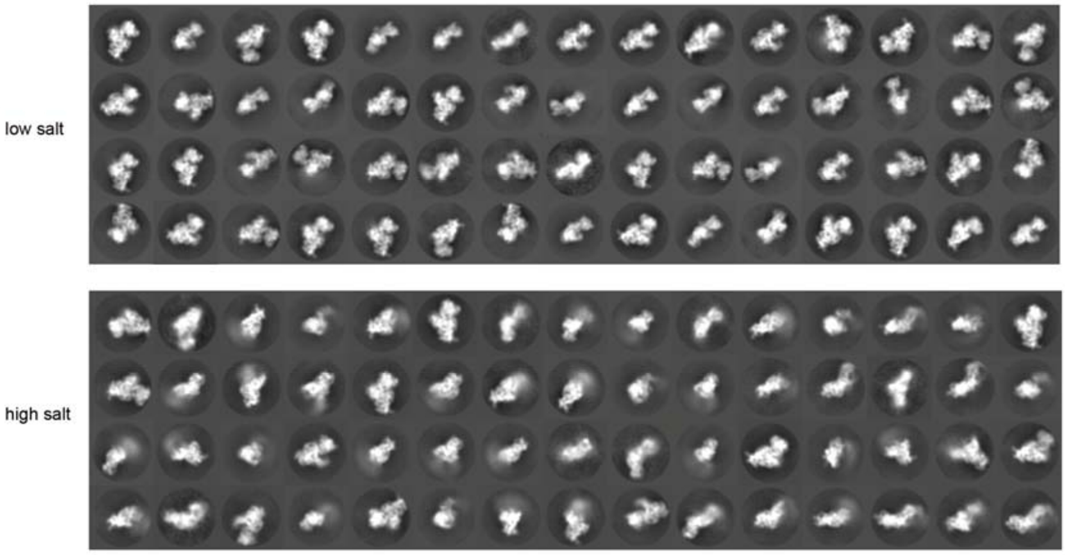

Supplemental Figure S1: 2D averages of the Δ*rbfA*Δ*rsgA* immature 30S particles prepared under low salt (A) and high salt (B) conditions. The reference-free classification was performed with RELION (Scheres, 2012).

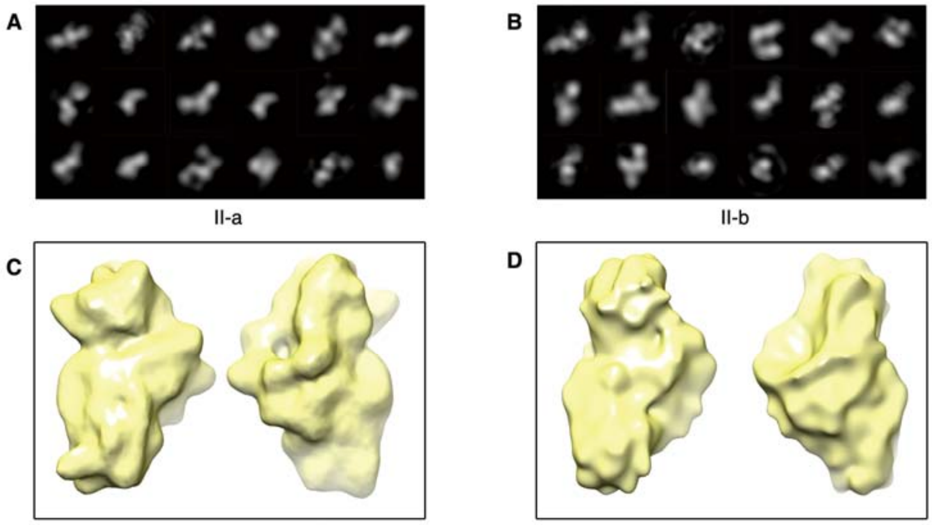

Supplemental Figure S2. Analysis of particles (II-a and II-b groups) from high salt treated sample at both 2D and 3D levels. Particles from the II-a and II-b groups were analyzed with RELION at both 2D (A and B) and 3D (C and D) levels. Maps are displayed from intersubunit and solvent views. Clearly, these two structures lack reliable structural details, and the resolutions of these two structures are also the worst (Supplemental Table S2). Reference-free classification (A and B) indicates they are mixtures of heterogeneous particles, probably including low quality particles and high salt-deformed particles, as well as very early-stage particles. Together, it indicates that the structures in (C and D) are simply biased global averages.

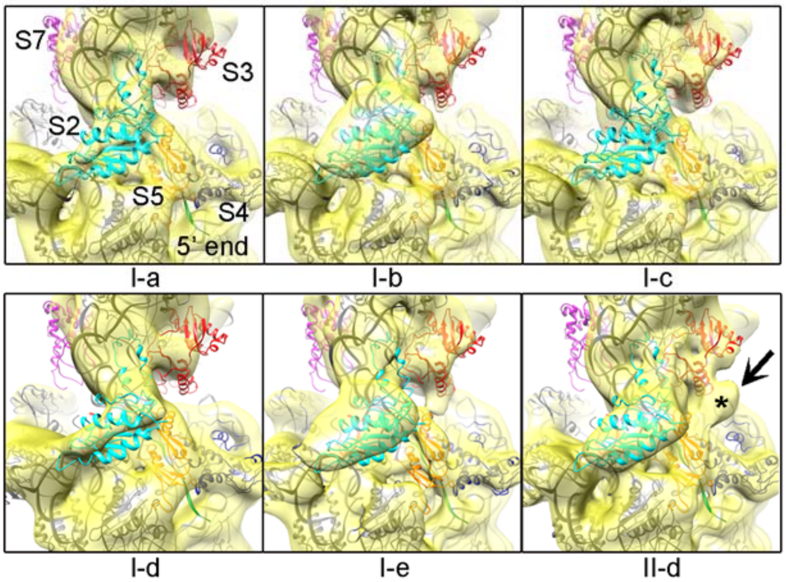

Supplemental Figure S3. Comparison of the late-stage structures from the low salt (I-a to I-e) and high salt (II-d) treated samples. Density maps are displayed at a comparable 3σ contour level, with the atomic model of the mature 30S subunit superimposed. S2, S3, S4, S5 and S7 are colored cyan, red, blue, orange and magenta, respectively. The extra density blob that is probably residual density for the 5' leader sequence of the 17S rRNA is denoted by an asterisk and an arrow in the map of II-d.

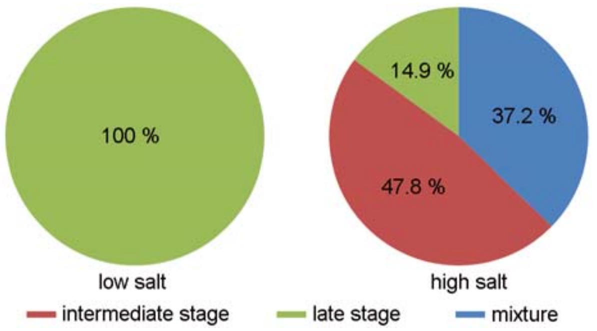

Supplemental Figure S4. Particle distributions of Δ*rbfA*Δ*rsgA* immature 30S subunits isolated under two different salt conditions. Intermediate stage particles (II-c and II-e) include particles with extremely mobile head domains; late stage particles (I and II-d) include particles with relatively rigid head domains; mixture particles (II-a and II-b groups) include low quality particles and high salt-deformed particles, as well as very early-stage particles.

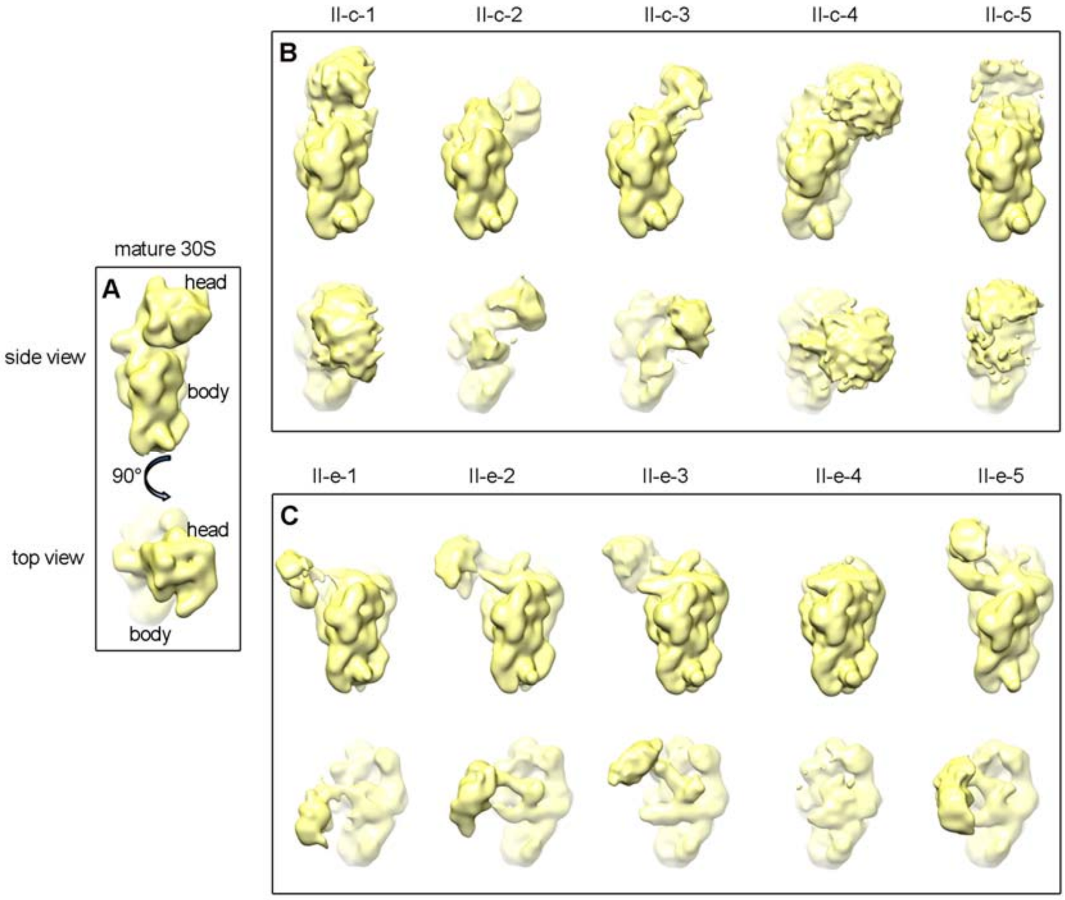

Supplemental Figure S5. Positional difference of the head domain in the subclass structures from the II-c and II-e groups. Density maps of the mature 30S subunit (A), subclasses of the II-c (B) and the II-e (C) groups are shown from the side (upper) and top (lower) views.

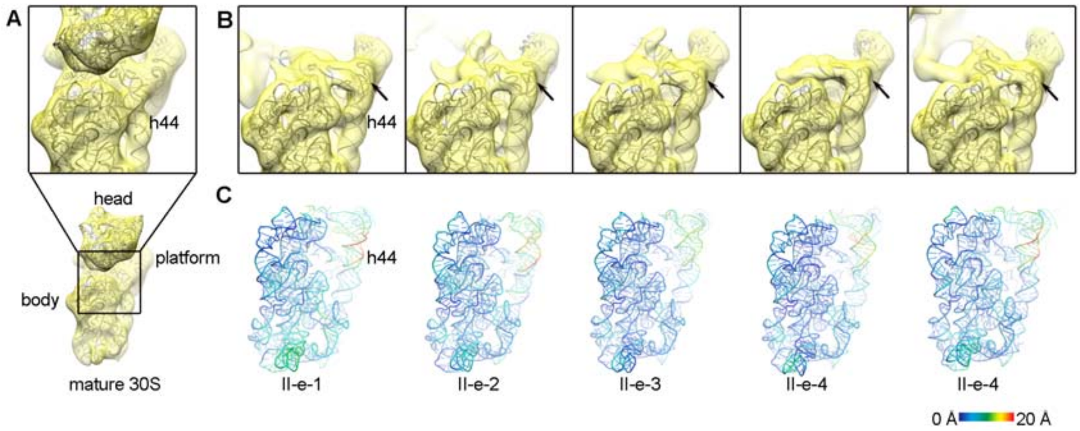

Supplemental Figure S6. Premature docking of h44 in the subclass structures of the II-e group. (A) The density map of the mature 30S subunit is displayed in transparent yellow, superimposed with the atomic model, and zoomed in a view showing the decoding center. (B) Density maps of subclasses from the II-e group are shown in the same view as (A), superimposed with their flexibly fitted atomic models. (C) Deviations of the body domain of 16S rRNA in these maps are shown as temperature maps. The black arrows in (B) indicate the upper part of h44, which is in a different conformation from that of the mature 30S subunit, indicating that the docking of h44 in these subclass structures is premature.

Supplemental References

Guo, Q., Goto, S., Chen, Y., Feng, B., Xu, Y., Muto, A., Himeno, H., Deng, H., Lei, J., and Gao, N. (2013). Dissecting the in vivo assembly of the 30S ribosomal subunit reveals the role of RimM and general features of the assembly process. Nucleic acids research 41, 2609-2620.

Jomaa, A., Stewart, G., Martin-Benito, J., Zielke, R., Campbell, T.L., Maddock, J.R., Brown, E.D., and Ortega, J. (2011). Understanding ribosome assembly: the structure of in vivo assembled immature 30S subunits revealed by cryo-electron microscopy. Rna 17, 697-709.

Leong, V., Kent, M., Jomaa, A., and Ortega, J. (2013). Escherichia coli rimM and yjeQ null strains accumulate immature 30S subunits of similar structure and protein complement. Rna 19, 789-802.

Scheres, S.H. (2012). A Bayesian view on cryo-EM structure determination. Journal of molecular biology 415, 406-418.
